# New blind species and new records of *Sinella* from Nanjing, China (Collembola, Entomobryidae)

**DOI:** 10.3897/zookeys.604.7902

**Published:** 2016-07-11

**Authors:** Heng Xu, Feng Zhang

**Affiliations:** 1 College of Plant Protection, Nanjing Agricultural University, 1 Weigang, Nanjing 210095, P. R. China

**Keywords:** Springtail, eyeless, Sinella quinseta sp. n., Sinella qixiaensis sp. n.

## Abstract

Two new blind species of *Sinella* are described from Nanjing, China. *Sinella quinseta* **sp. n.** from Purple Mountain possesses unique 5+5 central macrochaetae on Abd. II, and can be distinguished from other species of the genus by the postlabial chaetae and the dorsal chaetotaxy. *Sinella qixiaensis* **sp. n.** from Qixia Mountain is characterized by the paddle-like S-chaetae of Ant. III organ and the smooth straight chaetae on the manubrium and base of dens; it differs from two closely related species by the smooth manubrial chaetae, the labial chaetae, the Ant. III organ, and the macrochaetae on Abd. II. *Sinella fuyanensis* Chen & Christiansen and *Sinella quinocula* Chen & Christiansen were also newly recorded from Nanjing.

## Introduction

The genus *Sinella* has a worldwide distribution, and is particularly abundant in China. Deharveng (1990), Chen and Christiansen (1993) and Zhang et al. (2009, 2011) made main contributions to the modern taxonomy of this genus. To date, a total of 37 species, including 25 eyed (Ding and Zhang 2015) and 12 blind ones, have been reported from China. Among them, only four-eyed but no blind species were recorded from Nanjing, Jiangsu Province: *Sinella curviseta* Brook, 1882, *Sinella triocula* Chen & Christiansen, 1993, *Sinella affluens* Chen & Christiansen, 1993, and *Sinella quinocula* Chen & Christiansen, 1993. In the present paper, two new blind species and two new records are reported from Nanjing.

## Materials and methods

Specimens were cleared in Nesbitt’s fluid, mounted under a coverslip in Marc André II solution, and studied using a Nikon E80i microscope. The labial chaetae terminology follows Gisin’s system (1967). The dorsal and ventral chaetotaxy of head and the Ant. III organ are described after Chen and Christiansen (1993). Dorsal body chaetae are designated following Szeptycki (1979) and Zhang et al. (2011). The number of macrochaetae is given by half-tergite in the descriptions (left side of tergites drawn in figures). Tergal S-chaetotaxic formula follows Zhang and Deharveng (2015). All materials are deposited in the collections of the Department of Entomology, College of Plant Protection, Nanjing Agricultural University (NJAU), P. R. China.

### Abbreviations

Th.
 thoracic segment 


Abd.
 abdominal segment 


Ant.
 antennal segment 


mac
 macrochaeta/ae 


mic
 microchaeta/ae 


ms
 S-microchaeta/ae 


sens
 ordinary tergal S-chaeta/ae 


## Taxonomy

### Sinella quinseta sp. n.

Taxon classificationAnimaliaCollembolaEntomobryidae

http://zoobank.org/C83F07ED-4F4C-492D-A2B6-BBCCA5352450

Figs 1–14 , 15–18

#### Material.

Holotype: ♀ on slide, China, Jiangsu Province, Nanjing, Purple Mountain, Tomb of Liao Zhongkai and his wife He Xiangning, 32.056°N, 118.830°E, altitude 38 m, 10 April 2009, Feng Zhang and Daoyuan YU leg. (# C9581). Paratypes: 1 ♂ and 4 ♀♀ on slides and 5 juveniles in alcohol, same data as holotype.

#### Etymology.

Named after the unique 5+5 central mac on Abd. II in this new species.

#### Diagnosis.

No eyes. Two internal sens of Ant. III organ expanded. Long smooth straight chaetae absent on antennae. Clypeal chaetae 7(5). Postlabial chaetae X, X_2_ and X_4_ ciliate. No “smooth” inner differentiated tibiotarsal chaetae. Tenent hairs clavate. Manubrium without smooth chaetae. Tergal ms as 1, 0|1, 0, 0, 0. Abd. II with 5+5 central mac. Abd. IV with 5+5 central and 5+5 lateral mac.

#### Description.

Body length up to 1.17 mm. Body pale in alcohol.

**Figures 1–14.**
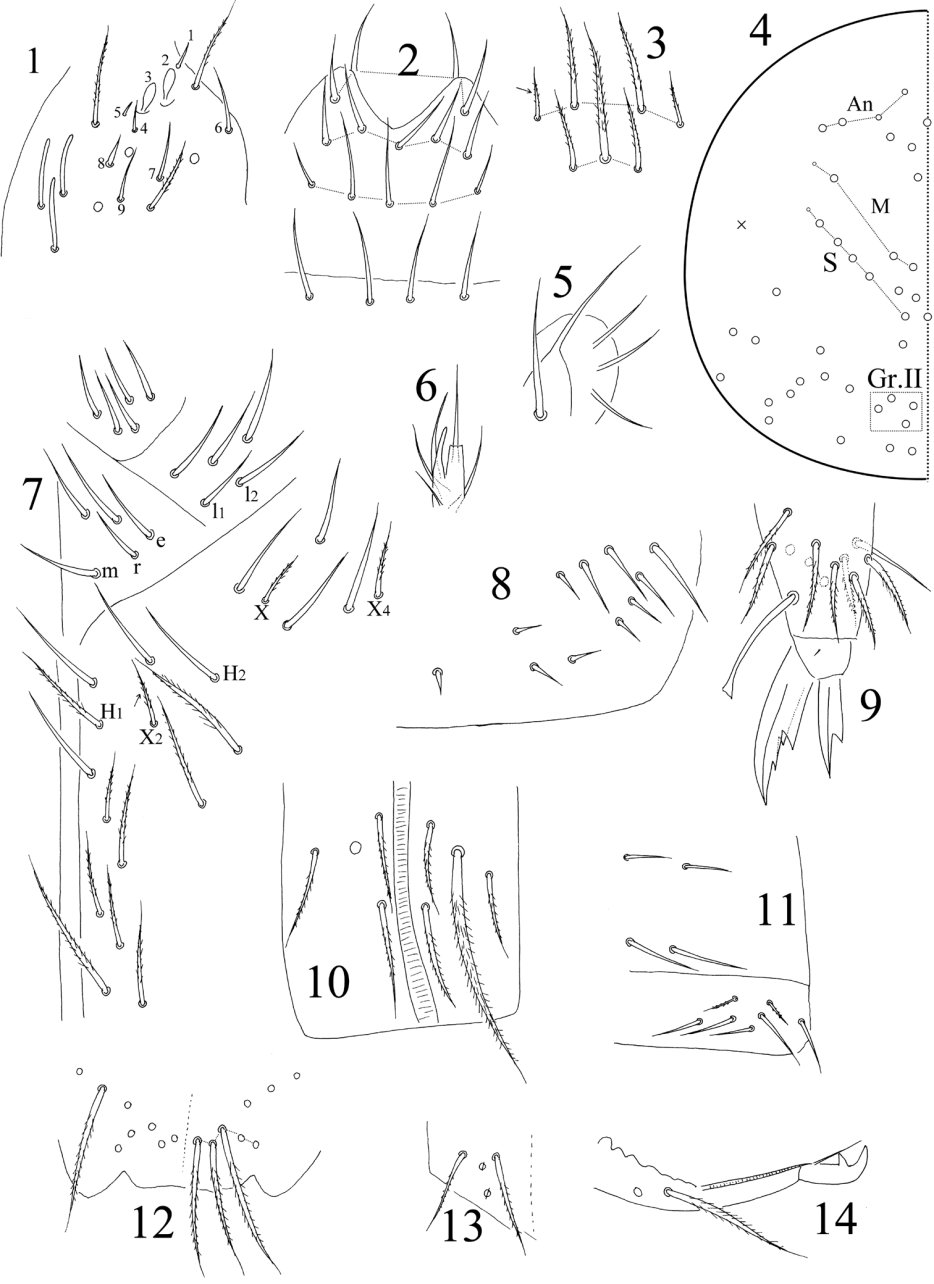
*Sinella quinseta* sp. n. **1** Ant. III organ **2** labrum **3** clypeal chaetae (arrow indicates that the chaeta may be absent) **4** dorsal cephalic chaetotaxy **5** maxillary outer lobe **6** lateral process of labial palp E **7** chaetae on the ventral side of head **8** trochanteral organ **9** hind claw **10** anterior face of ventral tube **11** ventral face and lateral flap of ventral tube **12** distal part of anterior face of manubrium **13** manubrial plaque **14** mucro.

Antenna 1.69–1.80 times as long as cephalic diagonal. Antennal segments ratio as I : II : III : IV = 1 : 2.00–2.17 : 1. 82–1.90 : 3.12–3.18. Smooth spiny mic at base of antennae 3 dorsal, 3 ventral on Ant. I, 1 internal, 1 external and 1 ventral on Ant. II. Ant. II distally with 1 rod-like S-chaeta. Two internal sens of Ant. III organ expanded (Fig. 1). Long smooth straight chaetae absent on antennae.

Eyes absent. Prelabral and labral chaetae 4/ 5, 5, 4, all smooth; the three median chaetae of the row a longer than lateral ones (Fig. 2). Labral papillae absent. Clypeal chaetae 7(5), arranged in two rows; the inner two chaetae of the anterior row of four chaetae smooth in one specimen; most lateral two small chaetae absent in two specimens (Fig. 3). Dorsal cephalic chaetotaxy with four antennal (An), three median (M) and five sutural (S) mac; Gr. II with four mac (Fig. 4). Mandible teeth 4+5. Subapical chaeta of maxillary outer lobe thicker than apical one and subequal in length; three smooth sublobal hairs on maxillary outer lobe (Fig. 5). Lateral process of labial palp slightly thicker than normal chaetae, with tip beyond apex of labial papilla E (Fig. 6). Labial chaetae as mrel_1_l_2_, all smooth, r/m = 0.60–0.68; chaetae X, X_2_ and X_4_ ciliate; X_2_ often absent; chaeta H_1_ ciliate; H_2_ smooth in one specimen and ciliate in others. Cephalic groove with 9(8) chaetae, 2(3) smooth and others ciliate (Fig. 7).

Trochanteral organ with 9–13 smooth spiny chaetae; 7–9 in arms and 2–4 between them (Fig. 8). Inner differentiated tibiotarsal chaetae ciliate with ciliations not closely appressed to axis. Tibiotarsi distally with ten chaetae in a whorl. Unguis with three inner teeth; two paired teeth unequal, outer one large. Unguiculus with a large outer tooth. Tenent hairs clavate (Fig. 9). Abd. IV 2.44–3.32 times as long as Abd. III along dorsal midline. Ventral tube anteriorly with 4–5 ciliate chaetae; one of them much larger than others (Fig. 10); posteriorly with 4 smooth chaetae; each lateral flap with 5 smooth and 2 ciliate chaetae (Fig. 11). Manubrium without smooth chaetae. Manubrium anteriorly with 5+5 ciliate chaetae in the most distal row (Fig. 12). Manubrial plaque with 2+2 pseudopores and 2+2 ciliate chaetae (Fig. 13). Distal smooth part of dens 1.34–1.85 times as long as mucro. Mucro bidentate with apical tooth longer than subapical one; basal spine long, nearly reaching tip of the apical tooth (Fig. 14).

**Figures 15–18.**
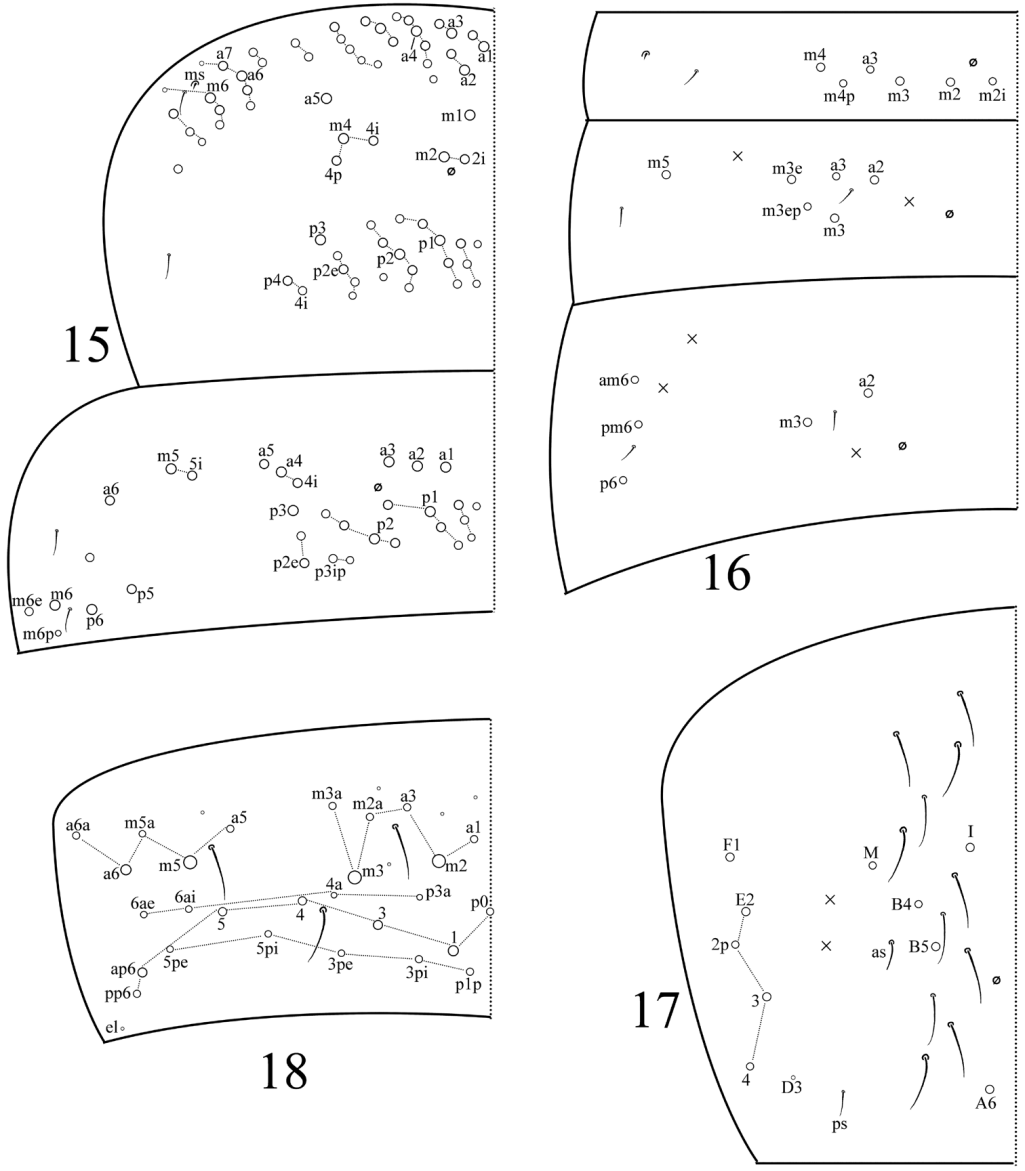
Tergal chaetotaxy in *Sinella quinseta* sp. n. **15** thorax **16** Abd. I‒III **17** Abd. IV **18** Abd. V.

Th. II with 3 (m1, m2, m2i) medio-medial, three (m4, m4i, m4p) medio-lateral, 20–22 posterior mac, one ms and two sens; ms inner to sens. Th. III with 29–32 mac and two lateral sens; mac a6i absent (Fig. 15). Abd. I with six (a3, m2–4, m2i, m4p) mac, one ms and one sens; sens inner to ms. Abd. II with five (a2, a3, m3, m3e, m3ep) central, one (m5) lateral mac and two sens. Abd. III with two (a2, m3) central, three (am6, pm6, p6) lateral mac and two sens; ms absent (Fig. 16). Abd. IV with five central (I, M, B4, B5, A6), five lateral mac (E2–4, E2p, F1), and approximately 13 sens; as and ps shorter than others (Fig. 17). Abd. V with three sens; chaeta p5a absent (Fig. 18).

#### Ecology.

In decomposing leaves along the roads.

#### Remarks.

*Sinella quinseta* sp. n. is characterized by blindness, ciliate postlabial chaeta H_1_ and 5+5 central mac on Abd. II. It is most similar to *Sinella yunnanica* Zhang & Deharveng, 2011 in being blind, its claw structure, the lateral process of labial palp, mucro, and chaetotaxy of head, thorax and Abd. IV, but differs from it in the presence of expanded internal S-chaetae on Ant. III organ, ciliate H_2_, X, X_2_ and X_4_ posterior to labium, 5+5 central mac on Abd. II, and the ventral tube.

### Sinella qixiaensis sp. n.

Taxon classificationAnimaliaCollembolaEntomobryidae

http://zoobank.org/DBFF3DC3-BC1D-4FC3-B5E3-5CB4E1D076E1

Figs 19–30 , 31–37

#### Material.

Holotype: ♂ on slide, China, Jiangsu Province, Nanjing, Qixia Mountain, 32.160°N 118.960°E, altitude 114 m, 6 December 2014, Daoyuan Yu and Chunyan Qin leg. (#14NJQX4). Paratype: 4 ♀♀ on slides and 5 in alcohol, same data as holotype.

#### Etymology.

Named after the type locality.

#### Diagnosis.

No eyes. Two internal sens of Ant. III organ paddle-like. Long smooth straight chaetae present on antennae. Clypeal chaetae eight. Postlabial chaetae X and X_2–4_ ciliate. “Smooth” inner differentiated tibiotarsal chaetae present. Tenent hairs I and II pointed or clavate, and III always clavate. Manubrium with smooth chaetae. Tergal S-microchaetae as 1, 0|1, 0, 0, 0. Abd. II with 4(3) central mac on each side. Abd. IV with 7+7 central and 6+6 lateral mac.

#### Description.

Body length up to 2.01 mm. Body pale in alcohol.

**Figures 19–30.**
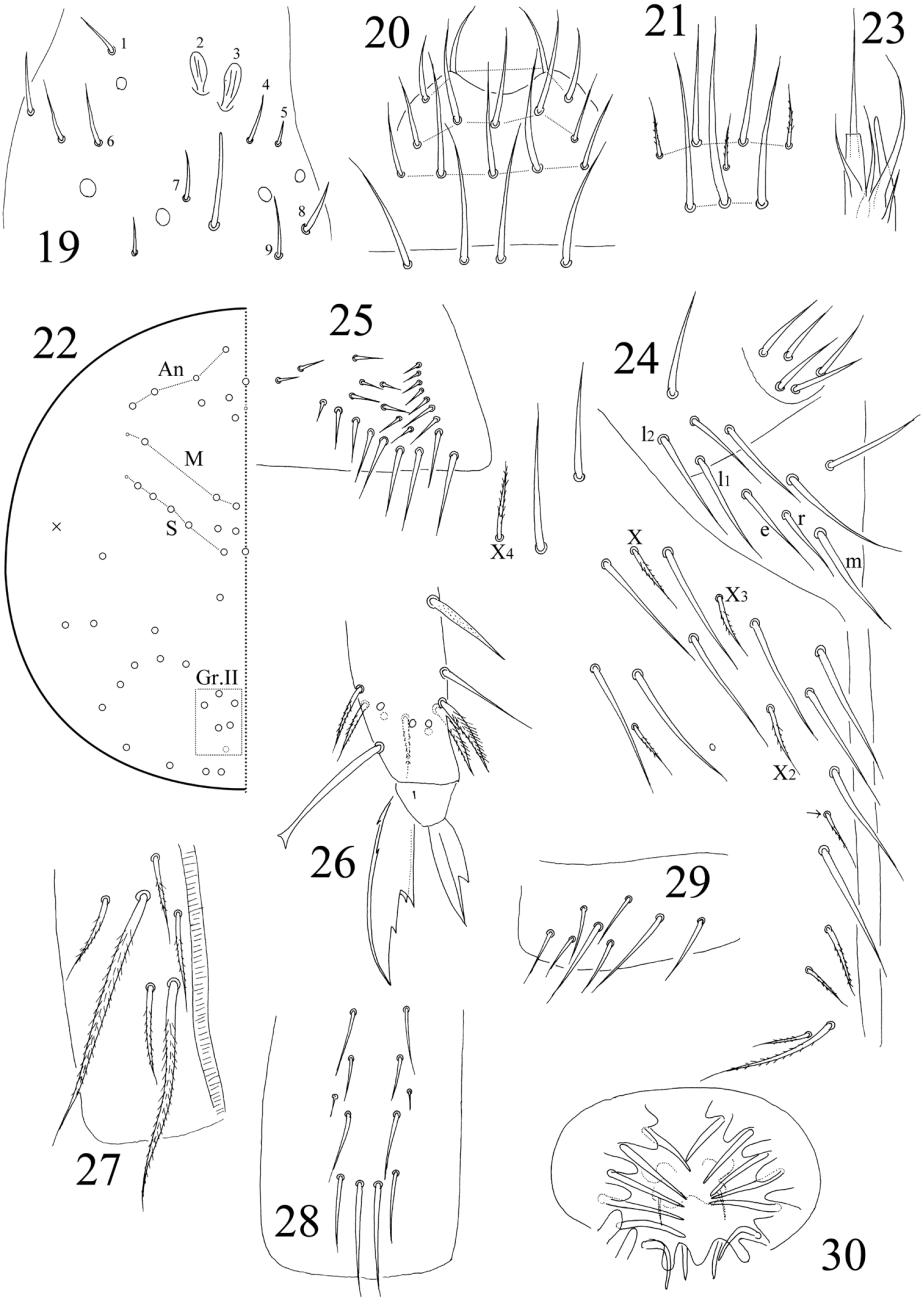
*Sinella qixiaensis* sp. n. **19** Ant. III organ **20** labrum **21** clypeal chaetae **22** dorsal cephalic chaetotaxy **23** labial palp **24** chaetae on the ventral side of head **25** trochanteral organ **26** hind claw **27–29** ventral tube **27** anterior face **28** posterior face **29** lateral flap **30** male genital plate.

Antenna 2.41–2.68 times as long as cephalic diagonal. Antennal segments ratio as I : II : III : IV = 1 : 1.66–1.96 : 1. 59–1.83 : 2.44–3.00. Smooth spiny mic at base of antennae three dorsal, three ventral on Ant. I, one internal, one external and two ventral on Ant. II. Ant. II distally with two rod-like sens. Two internal sens of Ant. III organ paddle-like (Fig. 19). Ant. IV with a knobbed subapical organ. Long smooth straight chaetae at least five ventral on Ant. I, at least 13 ventral on Ant. II, and one ventral on Ant. III.

Eyes absent. Prelabral and labral chaetae 4/ 5, 5, 4, all smooth; median three chaetae of the row a longer than lateral ones; labral intrusion not U-shaped (Fig. 20). Labral papillae absent. Clypeal chaetae eight in number, of which three are ciliated and small (Fig. 21). Dorsal cephalic chaetotaxy with four antennal (An), three median (M) and five sutural (S) mac; Gr. II with 5–6 mac (Fig. 22). Mandible teeth 4+5. Subapical chaeta of maxillary outer lobe larger than apical one; three smooth sublobal hairs on maxillary outer lobe. Lateral process of labial palp slightly thicker than normal chaetae, with tip beyond apex of labial papilla E (Fig. 23). Labial chaetae as mrel_1_l_2_, all smooth, r/m=0.61–0.76; chaetae X and X_2–4_ ciliate; chaeta X_3_ rarely absent. Cephalic groove with 8–9 chaetae, 4(5) of them smooth and others ciliate (Fig. 24).

**Figures 31–37.**
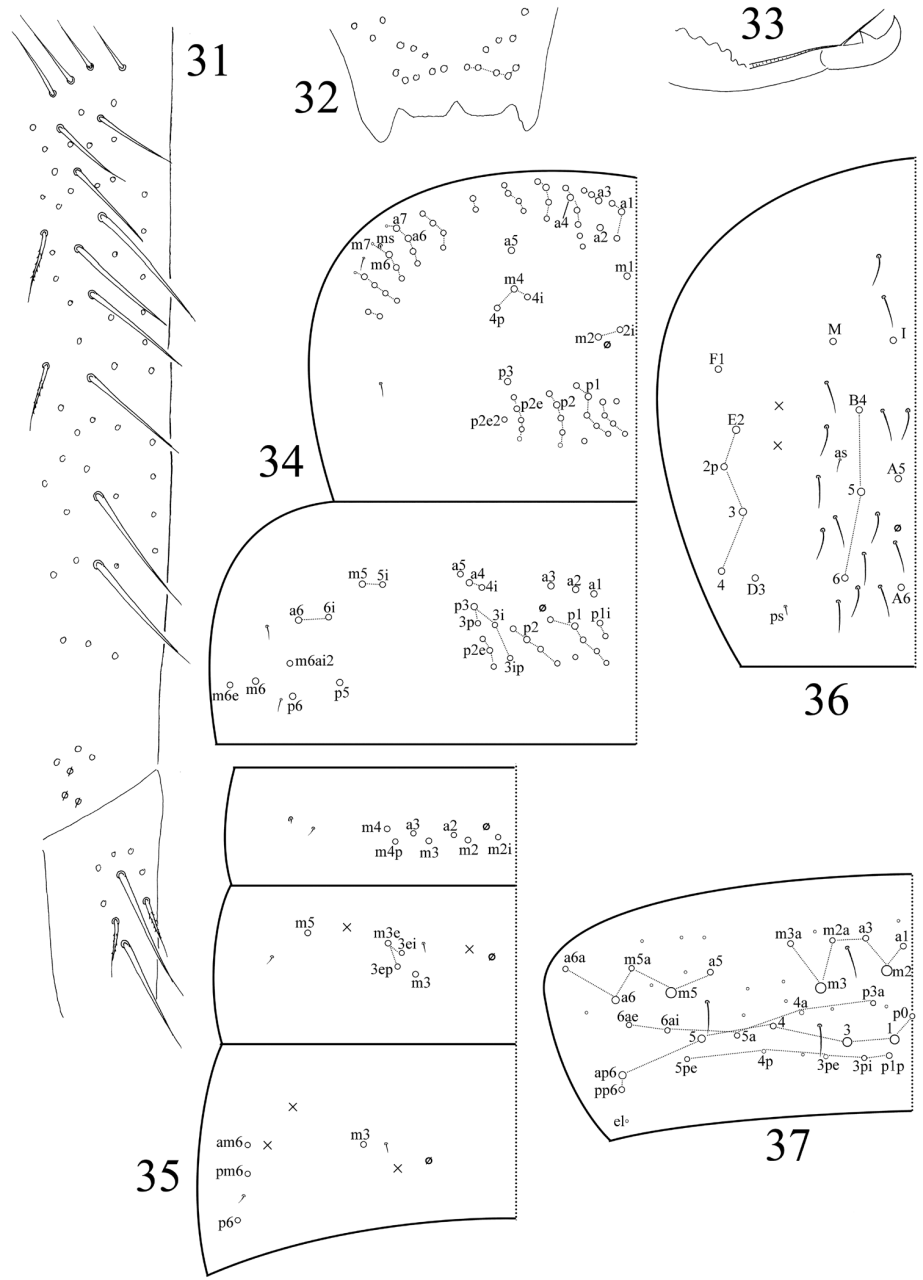
*Sinella qixiaensis* sp. n. **31** dorsal side of manubrium and base of dens **32** distal part of anterior face of manubrium **33** mucro **34–37** tergal chaetotaxy **34** thorax **35** Abd. I‒III **36** Abd. IV **37** Abd. V.

Trochanteral organ with approximately 24 smooth spiny chaetae; 13–15 in arms and 9–11 between them (Fig. 25). Partial inner differentiated tibiotarsal chaetae “smooth” with ciliations closely appressed to axis. Tibiotarsi distally with ten chaetae in a whorl. Unguis with three inner, one outer, and two lateral teeth; two paired inner teeth unequal, outer one large. Unguiculus with a large outer tooth. Tenent hairs I and II pointed or clavate, and III always clavate (Fig. 26). Abd. IV 3.13–4.67 as long as Abd. III along dorsal midline. Ventral tube anteriorly with 6+6 ciliate chaetae, two of them much larger than others (Fig. 27); posteriorly with 12 smooth chaetae (Fig. 28); each lateral flap with eight smooth chaetae (Fig. 29). Male genital plate with seven pairs of projections and internally with one pair of small chaetae (Fig. 30). Manubrium dorsally with about 13+13 smooth chaetae (Fig. 31); ventrally with 5+5 distal ciliate chaetae (Fig. 32). Manubrial plaque with 3+3 pseudopores and 3+3(2) ciliate chaetae. Base of dens with 2+2 smooth chaetae (Fig. 31). Distal smooth part of dens 1.04–1.12 as long as mucro. Mucro bidentate with apical tooth larger; basal spine long, with tip nearly reaching apical tooth (Fig. 33).

Th. II with three (m1, m2, m2i) medio-medial, three (m4, m4i, m4p) medio-lateral, 22–24 posterior mac, one ms and two sens; ms inner to sens. Th. III with 30–34 mac and two lateral sens; m5i, a6i, p5, p6, m6 and m6e as mac (Fig. 34). Abd. I with seven (a2–3, m2–4, m2i, m4p) mac, one ms and one sens; sens inner to ms. Abd. II with 4(3) (m3, m3e, m3ep, m3ei) central, one (m5) lateral mac and two sens; mac m3ei only absent on one side of one specimen. Abd. III with one (m3) central, three (am6, pm6, p6) lateral mac and two sens; ms absent (Fig. 35). Abd. IV with seven central (I, M, A5–6, B4–6), six lateral mac (D3, E2–4, E2p, F1), and at least 17 sens; sens as and ps short (Fig. 36). Abd. V with 3 sens (Fig. 37).

#### Ecology.

In decomposing leaves along the roads.

#### Remarks.

*Sinella qixiaensis* sp. n. is characterized by blindness, the paddle-like sens of Ant. III organ and abundant smooth chaetae on the manubrium. It is most similar to *Sinella insolens* Chen & Christiansen, 1993 and *Sinella sineocula* Chen & Christiansen, 1993. It differs from the former in the presence of smooth manubrial chaetae and the absence of labial chaeta M_1s_, and also differs from *Sinella sineocula* in the presence of smooth manubrial chaetae, the paddle-like sens of Ant. III organ, and the presence of mac m3ei on Abd. II.

### Sinella fuyanensis

Taxon classificationAnimaliaCollembolaEntomobryidae

Chen & Christiansen, 1993

Sinella (Sinella) fuyanensis Chen & Christiansen, 1993: 27. Type locality: China (Jiangxi).

#### Material.

♀ on slide and 4 in alcohol, China, Jiangsu Province, Nanjing, Lao Mountain, Long Cave, 32.051°N, 118.527°E, altitude 112 m, 10 April 2015, Daoyuan YU and Chunyan QIN leg. (# 15NJLS).

#### Ecology.

Known only from caves.

#### Distribution.

China (Jiangxi, Jiangsu).

### Sinella quinocula

Taxon classificationAnimaliaCollembolaEntomobryidae

Chen & Christiansen, 1993

Sinella (Sinella) quinocula Chen & Christiansen, 1993: 24. Type locality: China (Anhui).

#### Material.

♀ on slide and 5 in alcohol, China, Jiangsu Province, Nanjing, Lao Mountain, Longxing temple, 32.051°N, 118.527°E, altitude 112 m, 10 April 2015, Daoyuan YU and Chunyan QIN leg. (# 15NJLS).

#### Ecology.

Under stones.

#### Distribution.

China (Anhui, Jiangsu, Shaanxi).

## Supplementary Material

XML Treatment for Sinella quinseta

XML Treatment for Sinella qixiaensis

XML Treatment for Sinella fuyanensis

XML Treatment for Sinella quinocula
